# Cellular stress-induced up-regulation of FMRP promotes cell survival by modulating PI3K-Akt phosphorylation cascades

**DOI:** 10.1186/1423-0127-18-17

**Published:** 2011-02-13

**Authors:** Se Jin Jeon, Jung Eun Seo, Sung-Il Yang, Ji Woong Choi, David Wells, Chan Young Shin, Kwang Ho Ko

**Affiliations:** 1 Department of Pharmacology, College of Pharmacy and Research Institute of Pharmaceutical Sciences, Seoul National University, Seoul, Korea; 2 Molecular, Cellular and Developmental Biology, Yale University, New Haven, CT, USA; 3 Neuroscience Research Center, Institute for Biomedical Sciences and Technology, USA; 4 Department of Pharmacology, School of Medicine, Konkuk University, Seoul, Korea; 5 Department of Pharmacology, College of Pharmacy Gachon University of Medicine and Science, Incheon, Korea

## Abstract

**Background:**

Fragile X syndrome (FXS), the most commonly inherited mental retardation and single gene cause of autistic spectrum disorder, occurs when the Fmr1 gene is mutated. The product of Fmr1, fragile X linked mental retardation protein (FMRP) is widely expressed in HeLa cells, however the roles of FMRP within HeLa cells were not elucidated, yet. Interacting with a diverse range of mRNAs related to cellular survival regulatory signals, understanding the functions of FMRP in cellular context would provide better insights into the role of this interesting protein in FXS. Using HeLa cells treated with etoposide as a model, we tried to determine whether FMRP could play a role in cell survival.

**Methods:**

Apoptotic cell death was induced by etoposide treatment on Hela cells. After we transiently modulated FMRP expression (silencing or enhancing) by using molecular biotechnological methods such as small hairpin RNA virus-induced knock down and overexpression using transfection with FMRP expression vectors, cellular viability was measured using propidium iodide staining, TUNEL staining, and FACS analysis along with the level of activation of PI3K-Akt pathway by Western blot. Expression level of FMRP and apoptotic regulator BcL-xL was analyzed by Western blot, RT-PCR and immunocytochemistry.

**Results:**

An increased FMRP expression was measured in etoposide-treated HeLa cells, which was induced by PI3K-Akt activation. Without FMRP expression, cellular defence mechanism via PI3K-Akt-Bcl-xL was weakened and resulted in an augmented cell death by etoposide. In addition, FMRP over-expression lead to the activation of PI3K-Akt signalling pathway as well as increased FMRP and BcL-xL expression, which culminates with the increased cell survival in etoposide-treated HeLa cells.

**Conclusions:**

Taken together, these results suggest that FMRP expression is an essential part of cellular survival mechanisms through the modulation of PI3K, Akt, and Bcl-xL signal pathways.

## Background

Fragile X syndrome (FXS) is a well known neurodevelopmental disorder caused by loss of fragile X linked mental retardation protein (FMRP) which is encoded by Fmr1 gene [1]. FXS patients typically show a wide spectrum of cognitive and behavioral problems such as attention deficit, anxiety and mood disorder, increased risk of seizures, autistic spectrum behaviors, and mental retardation [1]. FMRP is expressed in many tissues including testis, placenta, and brain [2,3] and in a variety of cell types including HeLa [4].

FMRP is a RNA binding protein, which regulates translation of target mRNAs. A wide range of potential target mRNAs have been suggested, most of which have been correlated to the regulation of synaptic function as well as neuronal development (for a review, see [5,6]). Interestingly, many mRNAs encoding a diverse array of proteins having no known link to neuronal development and synaptogenesis were also suggested including phosphoinositide 3 kinase (PI3K) [7], amyloid precursor protein (APP) [8], and Bcl-2 interacting protein (Bnip) [9]. In addition, FMRP is found both in the nucleus and cytoplasm and shuttles between the two compartment depending on the cellular context [10,11], suggesting the cellular function of FMRP might be much broader than previously thought.

Recently, in a study using a Danish cohort of 223 patients with fragile X syndrome, Schultz-Pedersen and colleagues have reported that standardized incidence ratio (SIR) of cancer was reduced to 0.28 [12] compared with cancer rates in the general population, which can not be attributed to the compounding factors such as differences in mortality rate, neglected symptoms or failure in diagnosis etc. Although no clear mechanism of decreased cancer rates has been suggested in that particular study, it is one intriguing hypothesis that the lack of FMRP in FXS patients may alter cellular apoptosis/survival mechanism, thereby decreasing cancer incidence in the long run. In addition, investigations of proliferative stem cells from FMRP deficient mice or postmortem brain showed an increased number of TUNEL positive cells [13,14], which might suggest that the control of cellular survival mechanism is defective in FMRP deficient cells.

Regulation of Ras-PI3K-Akt signaling pathway is one of the essential regulators of cellular survival/apoptosis control and activated Ras-PI3K-Akt signaling was regarded as a hallmark of many cancer cells [15,16], which are characterized by unregulated apoptosis and prolonged survival. Akt/PKB is a serine/threonine protein kinase that plays a key role in multiple cellular processes such as cell proliferation, apoptosis, and transcription [17]. Generally, Akt increases cellular survival rates both directly and indirectly by mechanism involving the regulation of the level of (anti)apoptotic proteins such as Bcl-2 and Bcl-xL [17,18]. Collectively, Akt signaling pathway seems to be one of major mediators of cellular survival/death determinant. Interestingly enough, impaired PI3K-Akt activation in FXS was reported by Hu et al. [19], even though synaptic stimulation can induce upregulation of Ras activity.

In this study, using HeLa cells as a model system, which have been used for the elucidation of essential cellular functions of FMRP such as the association of FMRP in translating polysomes [20,21], biochemical interaction with the components of microRNA pathways [4,22,23] as well as translational inhibition of target mRNAs by FMRP [24,25], we tried to investigate the role of FMRP-PI3K-Akt pathway in the regulation of cell survival in the condition of etoposide-induced apoptosis.

Here we show that HeLa cells exposed to the cell death inducer etoposide up-regulate FMRP. This increase in FMRP synthesis was synchronized with the phosphorylation of Akt, a known cell survival-related signaling molecule. Indeed, cell survival was compromised when FMRP levels were reduced and was prolonged in cells over-expressing FMRP. Therefore, we provide the first experimental evidence that induction of FMRP plays a protective role against the stressed status of the cells.

## Methods

### Materials

Dulbecco's Modified Eagle's medium (DMEM), fetal bovine serum (FBS) and penicillin/streptomycin were purchased from Gibco-BRL (Grand Island, NY). The antibodies for Western blotting, p-PI3K, PI3K, p-Akt, and Akt were purchased from Cell Signaling (Beverly, MA). β-actin and Bcl-xL antibodies were from Santa Cruz Biotechnology (Santa Cruz, CA) and FMRP antibody was from Millipore (Billerica, MA). ECL™ Western blotting detection reagents were obtained from Amersham Life Science (Arlington Heights, IL). Trizol ^R ^reagents were purchased from Invitrogen (Carlsbad, CA). Small hairpin RNA virus (Sh RNA virus) associated reagents were all purchased from Sigma (St. Louis, MO, USA). SYBR green mix was obtained from Fermentas (Glen Burnie, Maryland) and TUNEL assay kit was obtained from Millipore (Billerica, MA). Transfection reagents were purchased from Invitrogen (Carlsbad, CA) and Roche (Roche Diagnostics Corp., Indianapolis, IN). All other reagents were purchased from Sigma (St. Louis, MO, USA). Dr. Darnell kindly provided eGFP- empty vector and eGFP-tagged FMRP vector.

### Cell culture and treatment

**Figure 1:**
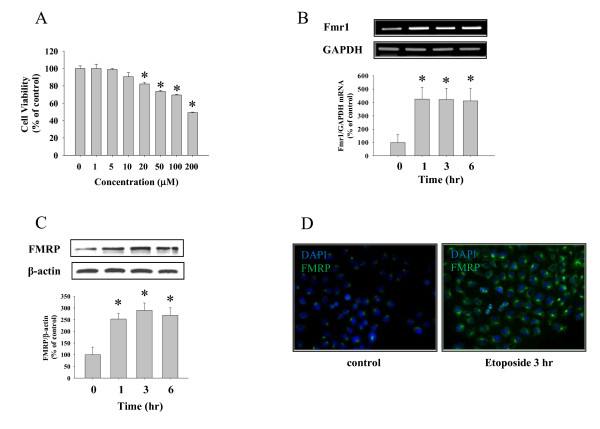
**Etoposide (ETO)-induced cell death and FMRP induction in HeLa cells**. (A) HeLa cells were treated with 1-200 μM ETO for 3 hr, then cell viability was analyzed by MTT assay. (B) *Fmr1 *mRNA level was analyzed by reverse transcription polymerase chain reaction (RT-PCR) procedures. For comparison, PCR reaction for housekeeping gene, GAPDH, was also performed. (C) The increase of FMRP protein was analyzed by Western blot and β-actin was used as a loading control. (D) ETO-induced expression of FMRP was visualized by immunocytochemistry. Blue fluorescence represents DAPI staining and green fluorescence means FMRP. Each graph represents quantification of RT-PCR and Western blot band intensity, respectively. Data represent mean ± S.E.M. * significantly different as compared with control and ^# ^significantly different as compared with ETO alone treatment (p < 0.01, n = 4).

HeLa cells were cultured in DMEM containing 10% heat-inactivated FBS, 100 units/ml penicillin and 100 μg/ml streptomycin for 2 days before treatment. Apoptosis-inducing conditions by etoposide were determined from preliminary experiments similar to those shown in Figure 1-A. Unless otherwise indicated, cells were challenged by treatment with 20 μM of etoposide for the indicated time period. The Akt inhibitor LY294002 (10 μM), Akt inhibitor IV (5 μM), and inhibitor VIII (10 μM) were added 1 hr prior to etoposide treatment. Before treatment, cells were washed and fresh serum free media was added. All cells were cultured at 37°C in humidified incubator containing 5% CO_2_.

### MTT assay

Cell viability was determined by MTT assay. After treatment, cells were incubated with the MTT solution (final concentration, 5 mg/ml) for 30 min. The dark blue formazan crystals formed in intact cells were solubilized with lysis buffer (100% ethanol) and the absorbance of samples was read at 540-595 nm with a microplate reader (Molecular Devices, Sunnylvale, CA, USA). Data were expressed as the percentage (%) of control (untreated cells).

### Western blot analysis

After washing with PBS two times, cells were lysed with 2X sample buffer (4% w/v SDS, 20% glycerol, 200 mM DTT, 0.1 M Tris-HCl, pH 6.8, and 0.02% bromophenol blue) and heated at 90°C for 10 min. The samples were then run through a 10% SDS-PAGE and transferred to nitrocellulose (NC) membrane. The NC membrane was blocked with 1 μg/ml polyvinyl alcohol (PVA) for 30 min at room temperature and incubated overnight at 4°C with the appropriate primary antibodies which were diluted at 1:5000 in 5% skim milk (Santa Cruz Biotechnology Inc., Santa Cruz, CA). After washing three times with PBS containing 0.2% Tween-20 (PBS-T), NC membranes were incubated with peroxidase-conjugated secondary antibodies for 2 hr at room temperature. After another three times washings, membranes were detected by enhanced chemiluminescence (Amersham, Buckinghampshire, UK).

### Reverse transcription polymerase chain reaction (RT-PCR)

Cells were washed with PBS and lysed using Trizol reagent (Invitrogen, Carlsbad, CA, USA) and extracted to total RNA according to the manufacturer's recommendation. 2 μg of total RNA was converted to cDNA by Maxime RT PreMix Kit (iNtRON Biotechnology, Seoul) and the amplification was performed using Maxime PCR premix Kit (iNtRON Biotechnology, Seoul). The procedure was consisted of 26 cycles (94°C, 1 min; 60°C, 1 min; 72°C, 1 min and continued by a final extension step at 72°C for 10 min) with the primers for FMRP (accession number NM_002024.4) and glyceraldehyde 3-phosphate dehydrogenase (GAPDH, accession number M17701). The following primers were used for amplification reactions:

for FMRP,

forward primer: 5'-TTG GTA CCT TGC ACA CAT CA-3'

reverse primer: 5'-AAG TTA GCG CCT TGC TGA AT-3'

for GAPDH,

forward primer: 5'-TCC CTC AAG ATT GTC AGC AA-3'

reverse primer: 5'-AGA TCC ACA ACG GAT ACA TT-3'

The expected size of the amplified DNA fragments was 486 base pairs for FMRP and 308 base pairs for GAPDH.

### Real time RT-PCR

Cells were extracted and mRNA converted to cDNA as above (RT-PCR). cDNAs were diluted at 1:10 in double distilled water and SYBR green mix. The PCR protocol was: 95°C for 30 sec, 60°C for 8 sec, 72°C for 15 sec, and continued by a final step at 4°C for 10 sec. After all the reactions were finished, data was compiled automatically by the equipment (Roche, Indianapolis, IN, USA).

### Immunocytochemistry (ICC)

HeLa cells on pre-coated cover glasses (Fisher Scientific, PA) were treated appropriately. After then, samples were washed twice and fixed with ice cold methanol (-20°C, 0.5 hr). For permeabilization, samples were incubated with permeabilization buffer (0.3% Triton X-100 in PBS) at room temperature for 15 min followed by blocking process using blocking buffer (1% BSA, 5% FBS in PBS). After 30 min, samples were incubated at 4°C overnight with an appropriate primary antibody (1:500 diluted at blocking buffer). Next day, after twice washing with diluted blocking buffer (1:10 diluted at PBS), samples were incubated with an appropriate secondary antibody (TMRE or FITC conjugated, 1:500 diluted at blocking buffer) at room temperature for 2 hr. Followed with three times of washing, samples were mounted using Vectashield (Vector laboratories, Burlingame, CA) and visualized with a fluorescence microscope (TCS-SP, Leica, Heidelberg, Germany) in randomly selected 5 areas.

### Small hairpin RNA (shRNA) virus preparation and transduction

#### (1) shRNA virus preparation

FMRP shRNA transfer vector were purchased as glycerol stocks (Sigma, SHGLY TRCN0000059759) and prepared using maxi-prep kit (QIAGEN, Valencia, CA, USA). After preparation, shRNA vector, packaging vector (Sigma) and FuGENE 6 (Roche) were mixed according to the manufacturer's recommendation and incubated with 293T cells for 24 hr. Next day, cells were replaced with fresh DMEM containing 10% FBS and incubated for another 24 hr. After 48 hr post-transfection, viral particles were collected by carefully removing the media and placing it in a collection tube. And the titer of viral particles was immediately determined by performing the HIV p24 Western blot assay and stored at - 70°C. The resulting shFmr1 virus targets Fmr1 gene (NM_002024) and the sequence composed of sense, loop, and antisense strands as follows:

CCGG**GCGTTTGGAGAGATTACAAAT**CTCGAG**ATTTGTAATCTCTCCAAACGC**TTTTT

As a control, non-target shRNA control vector was used (Sigma, SHC002) and its stem and loop structure is as follows:

CCGG**CAACAAGATGAAGAGCACCAA**CTCGAG**TTGGTGCTCTTCATCTTGTTG**TTTTT

#### (2) *in vitro *shRNA virus transduction

shRNA virus was used at 50 multiplicity of infection (MOI) for transduction. Briefly, cells were incubated with shRNA virus for 48 hr and replaced with fresh media. After recovery, cells were treated with etoposide as described in methods.

### Transfection of eGFP-tagged FMRP vector

Cells were transfected using Lipofectamine 2000 (Invitrogen, Carlsbad, CA) as suggested by the manufacturer with slight modification. A transfection cocktail consisting of 0.4 μg DNA and 2 μl lipofectmine was added to cells grown in 24 well plates containing opti-MEM media (GIBCO BRL, Grand Island, NY, USA). After 6 hr, the transfection cocktail was replaced with proper culture media and incubated for another 24 hr, followed by fresh media containing 0.4 μg/ml of G418. G418 media was replaced every 4 days to select cells expressing either eGFP or eGFP-FMRP. Cells were visualized using a fluorescence microscope as above (Leica, Heidelberg, Germany).

### Propidium iodide (PI) staining

HeLa cells plated on cover glass were treated as indicated above (20 μM of etoposide for 3 hr). After treatment, cells were incubated with propidium iodide (PI, 10 μg/ml, RNase 10 μg/ml) for 1 hr at room temperature followed by fixation using ice cold methanol for 30 min at -20°C and mounted using a Vectashield (Vector laboratories, Burlingame, CA). PI positive apoptotic cells were manually counted blind to experimental conditions in five visual fields which were chosen at random per each sample [26].

### TUNEL assay

After treatment, cells were fixed using 4% paraformaldehyde in PBS (pH 7.4) for 10 min at room temperature. Then samples were permeabilized by pre-cooled ethanol: acetic acid (2:1) for 5 min at -20°C. After two times wash, equilibration buffer was applied directly on the specimen and incubated for 10 sec at room temperature. Working strength TdT enzyme (70% reaction buffer+30% TdT enzyme) was added immediately and incubated in a humidified chamber at 37°C for 1 hr. The reaction was stopped for 10 min and samples were incubated with anti-digoxigenin-conjugated rhodamine in a humidified chamber for 30 min at room temperature. Specimens were mounted under a glass coverslip and observed as above.

### Fluocytometry (FACS) analysis

Following treatment, cells were harvested with Tris-EDTA buffer and centrifuged 13,000 rpm for 3 min at 4°C. Cells were suspended with 1 ml PBS and PI was added (2.5 μg/ml). Then samples were analyzed using FACS apparatus (BD Biosciences, San Jose, CA) for apoptotic cells.

### Data analysis

Data are expressed as the mean ± standard error of mean (S.E.M.) and analyzed for statistical significance by using one way analysis of variance (ANOVA) followed by Newman-Keuls test as a *post hoc *test and a p value < 0.01 was considered significant.

## Results

### Etoposide induced cell death and an increase in FMRP expression

To induce cell death in HeLa cells we used the topoisomerase II inhibitor etoposide. Preliminary experiments showed that other stimuli such as hydrogen peroxide and TNF-α□ produced similar cell death (data not shown) but etoposide gave the most consistent and robust response. Etoposide induced both concentration (Figure 1A) and time (data not shown) dependent cell death in HeLa cells as determined by MTT analysis. At 20 μM, etoposide also induced an increase in the steady state level of mRNA encoding FMRP (Figure 1B) as well as an increase in protein level of FMRP (Figure 1C-D) suggesting that etoposide treatment results in a transcriptional up-regulation of *Fmr1 *mRNA that leads to increased FMRP protein level.

In this study, the time window of Fmr1 mRNA and FMRP protein induction was 1-6 hr. To more specifically address the exact mechanism of protein induction in our system it might be needed to investigate earlier time points such as 15 and 30 min. Fmr1 mRNA is known for its translational control of protein synthesis by a rapid translation of pre-existing mRNA responding to stresses [27,28]. For example, after 15 min of light exposure, visual cortical FMRP expression was peaked at 30 min implying a post-transcriptional regulation of protein synthesis in this system [29]. Lim et al. suggested that the regulation of FMRP expression is highly modality-specific because either transcriptional or post-transcriptional mechanisms may modulate FMRP protein levels. Whether the translational control of Fmr1 mRNA may happen in HeLa cells at earlier time points after etoposide treatment may require additional experiments including the use of specific transcriptional and translational inhibitors.

### Akt phosphorylation is necessary for up-regulation of FMRP by etoposide

**Figure 2:**
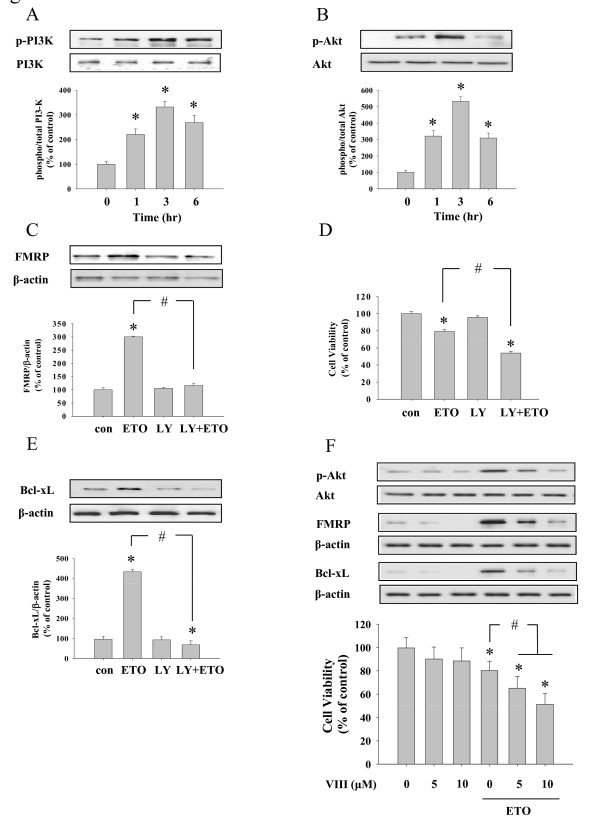
**Activation of PI3K and Akt pathway and its role in FMRP production and cell viability**. ETO-treated HeLa cells were analyzed using Western blot to investigate the activation of PI3K (A) and Akt (B). Western blot against total protein was used as a loading control. (C) An inhibitor of Akt phosphorylation, LY294002 (10 μM, LY) was pretreated and the level of FMRP was determined by Western blot. (D) After LY treatment, cell viability was measured by MTT assay, inhibition of Akt phosphorylation further decreased cell viability. (E) The level of BcL-xL after LY treatment. Increased BcL-xL expression induced by etoposide treatment was prevented by LY294002 treatment. (F) Another inhibitor of Akt, inhibitor VIII (0, 5, 10 μM, VIII) pretreatment also decreased activity and expression of Akt and Bcl-xL, respectively, in a concentration dependent manner. At the same time, cellular viability was also reduced by VIII treatment. The bar graphs represent the quantification of band intensity. Data represent mean ± S.E.M. * significantly different as compared with control and ^# ^significantly different as compared with ETO alone treated sample (p < 0.01, n = 4).

Akt (PKB) protein kinase is a critical regulator of many cellular functions including cell survival [30]. Therefore, we next investigated the activation state of the Akt signaling pathway after etoposide treatment, as indicated by the phosphorylation (activation) of PI3K and Akt. Etoposide induced an increase in phosphorylation of both PI3K (Figure 2A) and Akt (Figure 2B) within 1 hr of treatment. Since the PI3K-Akt pathway is a well known cellular pro-survival pathway [31], it was not surprising that inhibition of Akt phosphorylation by LY294002 increased cell death (Figure 2D) as well as the decrease in the level of Bcl-xL expression (Figure 2E) following etoposide treatment. However, interestingly, the up-regulation of FMRP protein by etoposide was also completely blocked by pretreatment with LY294002 (Figure 2C). This suggests that Akt phosphorylation is required for the induction of FMRP protein following etoposide stimulation.

To verify the role of Akt pathway in the regulation of FMRP induction and cell survival in etoposide treated cells, we used another inhibitor of Akt such as Akt inhibitor VIII (sc-202048, Santa Cruz Biotechnology, CA, USA) before etoposide treatment (Figure 2F). Akt inhibitor VIII reduced etoposide-induced phosphorylation of Akt as well as the induction of FMRP and Bcl-xL expression in a concentration dependent manner (Figure 2F, top panel). Akt inhibitor IV (sc-203809, Santa Cruz Biotechnology, CA, USA) also showed similar results (data not shown). Cellular viability was also decreased in Akt inhibitor VIII treated cells (Figure 2F, bottle panel). These results suggest that PI3K-Akt signaling pathway plays essential role in the control of FMRP-Bcl-xL expression and cell survival in etoposide treated HeLa cells.

### Loss of FMRP protein leads to an increase in etoposide-induced cell death

**Figure 3:**
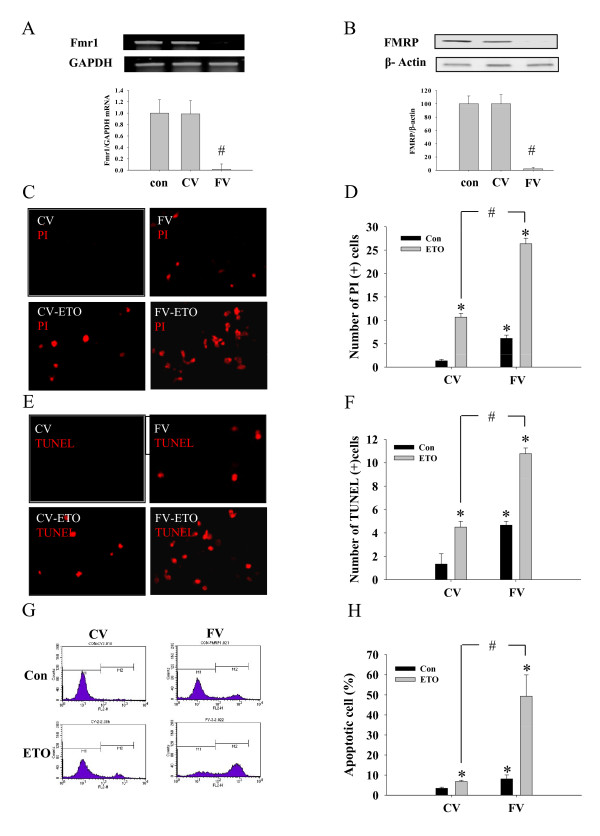
**sh*Fmr1 *virus silenced FMRP expression and reduced cellular viability in ETO treated HeLa cells**. To understand the role of FMRP on cellular viability, FMRP expression was inhibited by small hairpin RNA virus (sh*Fmr1 *lentivirus) as described in methods. RT-PCR band (A: top) and Real time PCR quantification (A: bottom) showed decreased level of *Fmr1 *mRNA. GAPDH was used as control. (B) Western blot assays was performed for FMRP and β-actin. (C) Apoptotic cell death after FMRP knock down. After sh*Fmr1*virus-induced inhibition of FMRP, HeLa cells were treated with ETO for 3 hr and then propodium iodide (PI) staining was performed. Red fluorescence represents PI positive cells. (D) Bar graph represents the quantification of the PI-positive apoptotic cells. (E) After an appropriate treatment as above, cells were stained with TUNEL as described. (F) Graph represents the quantification of the apoptotic cell number. (G) Histograms show the results of FACS analysis. (H) The graph represents the quantification of the apoptotic cells in FACS analysis. Data represent mean ± S.E.M. ^# ^Significantly different as compared with control (p < 0.01, n = 4), ^# ^significantly different as compared with control virus (CV) group (p < 0.01, n = 4). Con: control, CV: sh non targeted control virus and FV: sh*Fmr1 *virus.

To investigate the role of FMRP in the regulation of cell death, we first adopted loss of function experiments using sh*Fmr1 *viral transduction. After sh*Fmr1 *lentiviral transduction, the expression of both *Fmr1 *mRNA (Figure 3A) and FMRP (Figure 3B) was almost completely eliminated in HeLa cells. When we analyzed cell death using propidium iodide (PI) staining, cells transduced by sh*Fmr1 *virus (FV) but not control virus (sh non-targetd control virus, CV) showed increased cell death and also augmented etoposide-induced cell death at 3 hr after etoposide treatment (Figure 3C-D). To confirm PI staining results, apoptosis was further analyzed by using either TUNEL staining (Figure 3E-F) or FACS analysis (Figure 3G-H). The TUNEL positive cell number was increased 3.46 folds in basal FV compared to CV and etoposide treatment further increased the numbers of apoptotic HeLa cells (Figure 3E-F). In FACS analysis, the percentage of PI-positive apoptotic cell was much higher in FMRP knock-down condition (FV) both in basal (CV: 3.43 ± 0.57 and FV: 8.11 ± 1.99%) and etoposide-stimulated conditions (CV: 6.82 ± 0.62, and FV: 49.30 ± 10.65%) (Figure 3G-H).

**Figure 4:**
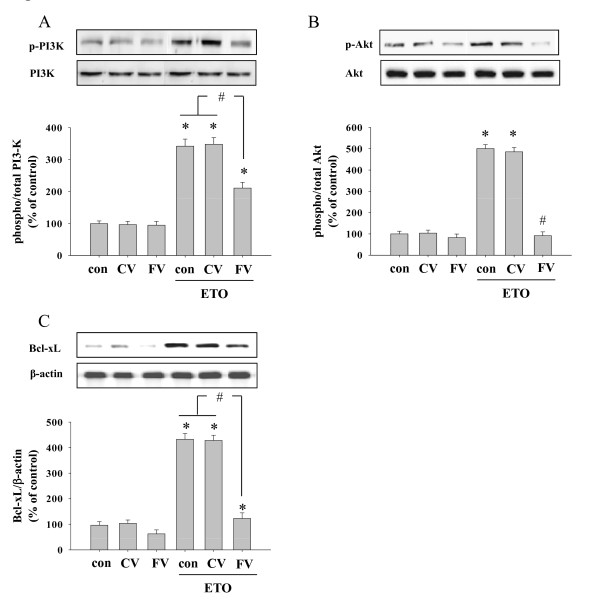
**Inhibition of Akt signaling pathway in FMRP knock-downed cells**. Cells were transduced with shRNA virus as mentioned above. After adaptation, cells were treated with ETO (20 μM, 3 hr). Then phosphorylated Akt signaling pathways were analyzed using Western blot assays. ETO-induced PI3K (A) and Akt (B) phosphorylation and Bcl-xL (C) expression were decreased compared with control in FMRP knock-downed HeLa cells. Graphs represent the densitometric quantification of the band intensity. * significantly different as compared with control, ^# ^significantly different as compared with control virus (CV) group (p < 0.01, n = 4). Con: control, CV: sh non targeted control virus and FV: sh*Fmr1 *virus.

To elucidate the mechanisms of cell survival regulation via FMRP, we next investigated the cellular signaling cascades (PI3K-Akt-Bcl-xL) in HeLa cells with artificially modulated FMRP level. The knock down of FMRP expression inhibited the ETO-induced activation of PI3K-Akt signaling cascades as compared to control condition (Figure 4A-B). Interestingly, etoposide-induced expression of Bcl-xL, a well known anti-apoptotic regulator, was also decreased 3.49 fold in FMRP knock down condition (Figure 4C). Taken together sh*Fmr1 *viral transduction showed an increase in apoptotic cell death after silencing of FMRP expression, which is resulted from deteriorated Akt signaling cascades (PI3K-Akt-Bcl-xL).

### Over-expression of FMRP protects HeLa cells against etoposide-induced apoptotic cell death

**Figure 5:**
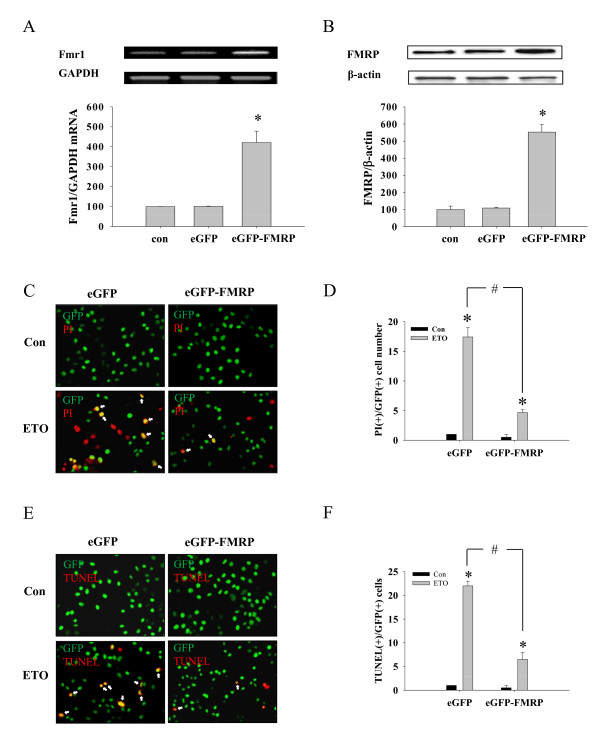
**Overexpression of eGFP-FMRP increased cell viability**. Cells expressing either eGFP- or eGFP-FMRP were lysed with Trizol for RT-PCR (A) to confirm *Fmr1 *level. As a loading control, GAPDH level was also analyzed. (B) Similarly, protein level was checked by Western blot. (C) Either eGFP- or eGFP-FMRP expressing cells were treated with ETO (20 μM, 3 hr). Then, cells were analyzed by PI staining. Red fluorescence represents PI positive cells and green fluorescence represents GFP positive cells. White arrows indicate yellow spots which represent double positive for PI and GFP. (D) Graphs represent the quantification of the PI-positive cell number. (E) Treated cells were stained with TUNEL as described above. Similarly, red fluorescence represents TUNEL positive cells and green fluorescence represents GFP positive cells. White arrows indicate yellow spots which represent double positive for TUNEL and GFP. (F) Bar graph represents the quantification of the number of the apoptotic cells. * significantly different from non treated cells and ^# ^significantly different as compared with eGFP transfected cells (p < 0.01, n = 4). eGFP denotes HeLa cells transfected with eGFP-empty vector, eGFP-FMRP indicates cells transfected with eGFP-FMRP vector.

To unequivocally demonstrate the role of FMRP on cell survival, we next over-expressed wild-type FMRP by using HeLa cells stably transfected with eGFP-tagged FMRP. The construction and use of these constructs were previously reported by the Darnell laboratory [32]. To confirm the transfection of eGFP-FMRP, we compared the level of *Fmr1 *mRNA (Figure 5A) and FMRP protein (Figure 5B) of these cells to untransfected HeLa cells by RT-PCR and Western blot, respectively. On average, cells stably transfected with eGFP-FMRP resulted in a 553.14% increase in FMRP protein over untransfected cells. Apoptosis, as determined by PI fluorescence staining, showed that cells over-expressing FMRP were less sensitive to etoposide-induced cell death (Figure 5C-D). Compared with eGFP-FMRP over-expressing cells, eGFP-empty vector expressing cells showed 3.76 ± 1.12 fold more PI positive cells, suggesting over-expression of FMRP decreased etoposide-induced cell death. This result was confirmed by quantifying TUNEL staining at 3 hr following etoposide treatment (Figure 5E-F). Similar protective effects of the over-expression of FMRP on ETO-induced cell death were also observed in TUNEL staining experiments (Figure 5E-F). Taken together, these data indicate that induction of FMRP has a protective role in the cell and delays the onset of apoptosis by etoposide on HeLa cells.

**Figure 6:**
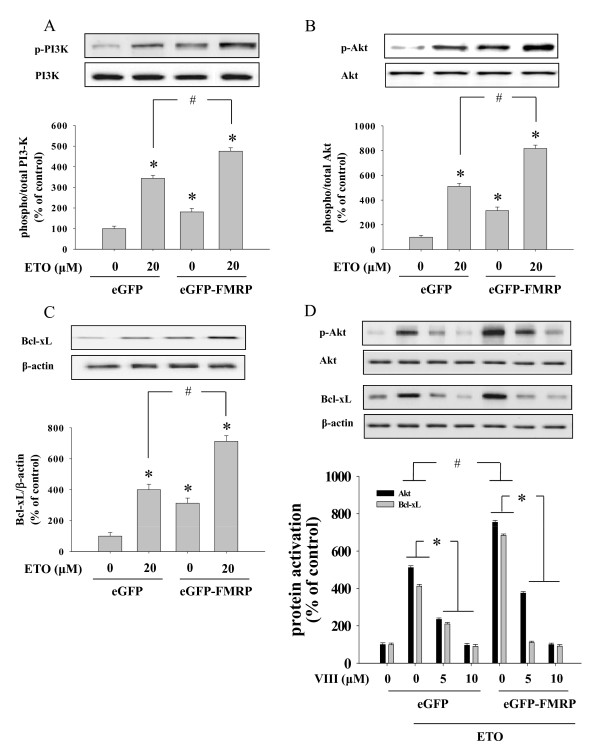
**The potentiation of PI3K-Akt phosphorylation and Bcl-xL expression by the overexpression of FMRP**. Cells transfected with wither eGFP- or eGFP-FMRP were treated with ETO (20 μM, 3 hr) and analyzed using Western blot. ETO-induced PI3K (A) and Akt (B) phosphorylation as well as Bcl-xL (C) expression were potentiated as compared with control. * significantly different as compared with control, ^# ^significantly different as compared with eGFP transfected cells. (D) A specific Akt inhibitor VIII reduced activation of Akt as well as induction of Bcl-xL in a concentration dependent manner. Graphs represent the densitometric quantification of the band intensity. * significantly different as compared with ETO treated cells, ^# ^significantly different as compared with eGFP transfected cells (p < 0.01, n = 4). eGFP denotes HeLa cells transfected with eGFP-empty vector, eGFP-FMRP indicates cells transfected with eGFP-FMRP vector.

Also activation of PI3K-Akt-Bcl-xL within cells harboring eGFP-FMRP vector was reinforced by etoposide treatment compared to eGFP-empty vector containing cells (Figure 6A-B). Also Bcl-xL induction was strengthened in case of FMRP over-expressed cells by 1.78 times (Figure 6C).

To validate the involvement of Akt activation in cell protection from etoposide-mediated apoptosis through the induction of FMRP, we used a specific Akt inhibitor VIII before etoposide treatment in eGFP-empty vector and eGFP-FMRP transfected HeLa cells.

As shown in figure 6D, etoposide-induced Akt phosphorylation was inhibited in a concentration dependent manner even in cells transfected with eGFP-FMRP. As expected, the increased expression of Bcl-xL by eGFP-FMRP was abolished by the treatment of Akt inhibitor VIII (Figure 6D), suggesting the essential role of Akt pathway in FMRP induced upregulation of Bcl-xL.

**Figure 7:**
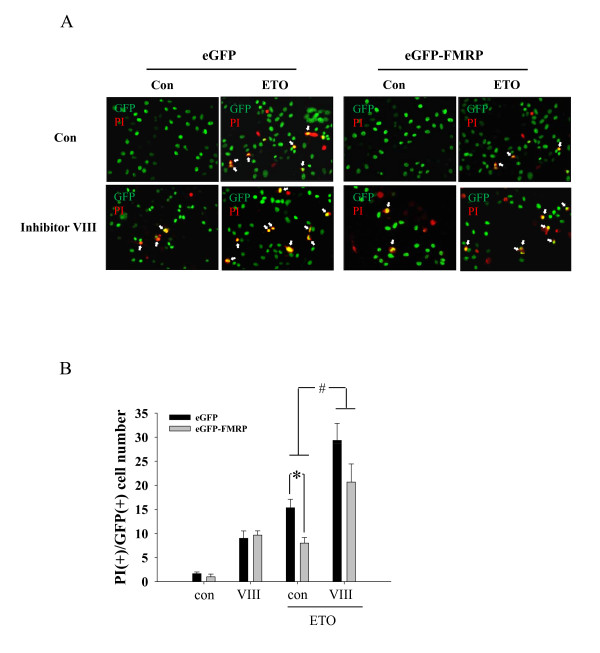
**Akt activation is necessary for the pro-survival effects of FMRP overexpression in ETO treated HeLa cells**. Cells transfected with wither eGFP- or eGFP-FMRP were incubated with Akt inhibitor VIII (10 μM, 1 hr) before ETO (20 μM, 3 hr) treatment and analyzed by PI staining. (A) Red fluorescence represents PI positive cells and green fluorescence represents GFP positive cells. White arrows indicate yellow spots which represent double positive for PI and GFP. (B) Graphs represent the quantification of the PI/GFP double-positive cell number. * significantly different as compared with eGFP transfected cells, ^# ^significantly different as compared with ETO treated HeLa cells (p < 0.01, n = 4). eGFP denotes HeLa cells transfected with eGFP-empty vector, eGFP-FMRP indicates cells transfected with eGFP-FMRP vector.

In addition, the decreased etoposide-induced apoptotic cell death in eGFP-FMRP transfected cells compared with eGFP transfected cells was prevented by Akt inhibitor VIII as determined by the quantification of the number of PI positive cell (Figure 7). Altogether, these results imply that Akt activation is an essential mediator of FMRP-mediated cellular survival response in stressed condition in HeLa cells.

Considering these results, we assume FMRP activates Akt signaling pathway, alleviates cellular stress, and ultimately, promotes cellular survival exposed to etoposide on HeLa cells.

## Discussion

Until now, FMRP has been implicated in various neurological diseases such as genetic FXS, autism, epilepsy, and attention deficit/hyperactivity disorder (ADHD). As such, research efforts have been focused on understanding FMRP function during development and the regulation of synaptic protein expression. However, even in these relatively intensely studied fields the exact regulatory mechanism(s) and function(s) of FMRP have yet to be fully elucidated.

In the work presented here, we describe for the first time a novel function for FMRP; that of a pro-survival protein. Using loss- and gain- of functional analysis, we determined that FMRP promotes cell survival under control conditions as well as upon the induction of cellular stress by the application of etoposide. Although the physiological significance of the present finding is not clear yet, the pro-survival nature of FMRP may suggest a role for FMRP being highly conserved and existence in several tissues. Interestingly, Fmr4, a non-coding RNA transcript in the FMR family, markedly affected human cell proliferation in vitro [33]. The knockdown of Fmr4 using siRNAs resulted in alteration of the cell cycle and increased apoptosis, while the over-expression of Fmr4 caused an increase in cell proliferation [33]. In that same study, a modest but significant decrease in proliferation of HeLa cells but not that of HEK 293T cells was also observed after siRNA induced silencing of *Fmr1*.

Also investigations from proliferative stem cells which were deficient in FMRP expression showed an increased number of TUNEL positive cells [13,14], which might be suggestive of increased programmed cell death in FMRP deficient cells [14].

FMRP has been linked to RNA interference silencing complex (RISC), a master of mRNA translation regulation, and shown to associate with the pool of mRNAs that eventually aggregate in stress granules upon cellular stress [34]. Indeed, Didiot reported that a loss of FMRP alters stress granule formation in puromycin- or arsenite-induced stress [34]. FMRP-containing mRNPs are dynamic structures that oscillate between polyribosomes and cytoplasmic granules similar to stress granules that contain repressed mRNAs in several cell types including HeLa [35]. The role of FMRP in stress granule formation and its role in cell viability following etoposide treatment have not been examined yet, however we feel these support our finding that FMRP plays a role in cell survival and suggest the possible function that in part may be mediated by harboring mRNAs in stress granules to overcome the damage.

In FXS patients, impaired Ras-PI3K signaling [19,36] and decreased cancer incident rate (0.28) [12] were reported compared with normal subjects. These show that a loss of FMRP results in a decrease in activated Ras-PI3K-Akt signaling pathway and a dysregulation of mTOR pathway, a well known target for FXS [37,38]. And our data implicate Akt activation in the induction of FMRP as a protective mechanism. In fact, many reported alteration of PI3K, Akt, and GSK3-β phosphorylation status in cells from FMRP knock-out patients and/or animals compared to wild-type [19,39]. Slightly different from our results, Gross suggested excess PI3K synthesis from FMRP knock-out animals was mediated through altered metabotropic glutamate receptor mediated control [40]. In addition, p110 subunit of PI3K and its upstream activator PI3K enhancer PIKE, two predicted targets of FMRP, are upregulated in FMRP knock-out mice [37]. However, p85 subunit of PI3K showed little or no changes in knock-out mice although there is a slight decrease in phosphorylation of PTEN at Ser380/Thr382/383, which may contribute to the inhibition of PI3K activity. In addition, the PI3K activity in FMRP knock-out did not show further increase of phosphorylation status exposed to DHPG (a selective group I mGluRs agonist) treatment [39,41], suggesting the aberration of stimulation-induced activation in FMRP knock-out animals [39,42] as observed in the present study. The discordance among groups including us may be explained in different experimental conditions such as origin, state, and stimulation conditions of cells.

FMRP also has been reported to be able to regulate Bcl-2 family expression or activation [9]. Among them Bcl-xL plays a pro-survival role against BAD actions [43-45] and many attempted to modulate it for cure and therapeutic means of cancer cell research [44,46]. In accordance with those studies, we focused on Bcl-xL as a molecular target of survival mechanism mediated by FMRP on etoposide-stimulated HeLa cells. Interestingly, FMRP controlled expression of Bcl-xL in etoposide-stimulated HeLa cells, which might be regulated by PI3K-Akt-mediated system (our data and [47]). Actually Bcl-xL expression was controlled by actions of transcription factors such as STAT, NF-kB, and FOXO family of transcription factors and signaling pathways of activated Ras, integrin, vitronectin, and hepatocyte growth factor [48]. Attractive points are among them STAT protein nuclear translocation [9], RAS, and integrin protein signal transduction [7], which were reported to be modulated by FMRP expression. So it seems possible that altered Bcl-xL expression in our study might be somehow affected by the existence of FMRP. In most cases, PI3K-Akt pathway delivers the pro-survival signal in many cell types [49]. Growth factors and integrins activate Akt by phosphorylation at Ser473 and Thr308, which leads to the modulation of Bad, caspase 9, and transcription factors including forkhead family and NF-kB [50]. Also Ramljak showed functional Akt activity was necessary for Bcl-xL expression in a series of experiments using dominant negative Akt vector and Akt inhibitor (LY294002) in epithelial cells [50]. Without Akt activity, cellular Bcl-xL expression was decreased significantly so the authors suggested Akt activation was obligatory for Bcl-xL regulation systems [50].

In etoposide-treated HeLa cells, regulation of apoptosis by Bcl-2 over-expression was reported [51]. Elliott showed pro-apoptotic members of Bcl-2 family, Bax and Bak expression was inhibited and also caspase was inactivated by Bcl-2 over expression, which led to the interruption of cell death. Like Bcl-2, the data from the present study may suggest that upregulation of Bcl-xL by FMRP plays anti-apoptotic roles in ETO treated HeLa cells.

In addition, our data suggested a possibility of a feed forward relationship between FMRP expression and Akt activation, in that FMRP was required, at least in part, for Akt phosphorylation and then activated Akt further promoted FMRP synthesis response in etoposide-stimulated condition. To strengthen this, we observed a decrease of FMRP levels in R25C transformed P19 cells which harbor a single amino acid switch (arginine into cysteine) in the 25th residue within Akt pleckstrin homology (PH) domain necessary for activation (data not shown). The role of Akt in the up-regulation of FMRP and the action of FMRP in the PI3K-Akt pathway activation may underlie the strong regulatory effects of FMRP in several physiological functions including synaptic plasticity (previous studies) and cellular survival (the present study).

## Conclusions

FMRP is abundant in a wide range of cell types and conserved through evolution; however, the exact role(s) are poorly elucidated in most cell types with the exception of the role FMRP plays in the regulation of structural and functional synaptic plasticity. In this study, we observed a robust FMRP induction after cell-death inducing stimuli in HeLa cells. Adopting molecular biology technology, we induced FMRP silencing and over-production, which showed detrimental and protective effects on cell viability, respectively, in both basal and etoposide-stimulated condition. The pro-survival role of FMRP may provide further insights into the role of FMRP in the regulation of cellular apoptosis from damaged as well as basal status.

## Competing interests

The authors declare that they have no competing interests.

## Authors' contributions

Se Jin Jeon participated in study design and conceptualization, analyzed data, and wrote the manuscript. Jung Eun Seo performed experiment and helped with analyzing data. David G. Wells participated in discussion of this study. Chan Young Shin conceptualized and designed the study and participated in writing and revision process. Kwang Ho Ko contributed study design and revised the manuscript for intellectual content. All authors read and approved the final manuscript.
